# Activation of the Extracellular Signal-Regulated Kinase Signaling Is Critical for Human Umbilical Cord Mesenchymal Stem Cell Osteogenic Differentiation

**DOI:** 10.1155/2016/3764372

**Published:** 2016-02-16

**Authors:** Chen-Shuang Li, Zhong Zheng, Xiao-Xia Su, Fei Wang, Michelle Ling, Min Zou, Hong Zhou

**Affiliations:** ^1^Key Laboratory of Shaanxi Province for Craniofacial Precision Medicine Research, College of Stomatology, Xi'an Jiaotong University, Xi'an, Shaanxi 710004, China; ^2^Department of Orthodontics, College of Stomatology, Xi'an Jiaotong University, Xi'an, Shaanxi 710004, China; ^3^Division of Growth and Development and Section of Orthodontics, School of Dentistry, University of California, Los Angeles, CA 90095, USA; ^4^University of Chicago, Chicago, IL 60637, USA

## Abstract

Human umbilical cord mesenchymal stem cells (hUCMSCs) are recognized as candidate progenitor cells for bone regeneration. However, the mechanism of hUCMSC osteogenesis remains unclear. In this study, we revealed that mitogen-activated protein kinases (MAPKs) signaling is involved in hUCMSC osteogenic differentiation* in vitro*. Particularly, the activation of c-Jun N-terminal kinases (JNK) and p38 signaling pathways maintained a consistent level in hUCMSCs through the entire 21-day osteogenic differentiation period. At the same time, the activation of extracellular signal-regulated kinases (ERK) signaling significantly increased from day 5, peaked at day 9, and declined thereafter. Moreover, gene profiling of osteogenic markers, alkaline phosphatase (ALP) activity measurement, and alizarin red staining demonstrated that the application of U0126, a specific inhibitor for ERK activation, completely prohibited hUCMSC osteogenic differentiation. However, when U0126 was removed from the culture at day 9, ERK activation and osteogenic differentiation of hUCMSCs were partially recovered. Together, these findings demonstrate that the activation of ERK signaling is essential for hUCMSC osteogenic differentiation, which points out the significance of ERK signaling pathway to regulate the osteogenic differentiation of hUCMSCs as an alternative cell source for bone tissue engineering.

## 1. Introduction

Although bone tissue has a high regenerative capacity, local endogenous cell numbers are often not adequate enough to reestablish tissue continuity or function in critical-sized defects [1–3]. Thus, there is a worldwide competition to develop engineered bone tissues to conquer this difficulty. However, after more than three decades of investigation, the success of bone tissue engineering is still limited [4]. One of the most critical obstacles is finding a suitable progenitor cell source. To date, human bone marrow mesenchymal stromal cells (hBMSCs) have been considered a native cell source and have been widely studied for osteogenic differentiation [5–7]. However several disadvantages, such as long derivation times, heterogeneous cell population, and variable potency [8, 9], markedly hinder the clinical application of hBMSCs for bone tissue engineering. Thus, alternative cell sources for bone tissue engineering are in high demand.

Human umbilical cord mesenchymal stem cells (hUCMSCs) isolated from Wharton's jelly of the umbilical cord have similar surface marker expression, high differentiation potential, low immunogenicity, and low tumorigenic risk as hBMSCs [10–17]. In contrast to hBMSCs that have to be harvested through invasive bone marrow aspiration, hUCMSCs are isolated from generally discarded tissue, umbilical cords, without ethical concerns [16, 18, 19], potential pain, and medical or surgical risks, such as bleeding and anesthesia [20]. Additionally, unlike hBMSCs and other stem cells isolated from adults, hUCMSCs share a high expansion capacity with fetal-derived stem cells [21]. These previous studies suggest that hUCMSCs may be a more suitable progenitor cell source than hBMSCs in a clinical setting [22], and thus multiple independent research groups have recruited hUCMSCs for various tissue regeneration, including bone tissue engineering [23–28]. However, the molecular mechanism of hUCMSC osteogenic differentiation has not been uncovered until now.

Mitogen-activated protein kinases (MAPKs) are a widely conserved family of serine-threonine protein kinases, including extracellular signal-regulated kinases (ERK), c-Jun N-terminal kinases (JNK), and p38 [29]. Previous studies have indicated that distinct MAPK pathways independently modulate stem cell self-renewal and differentiation [30, 31]. For instance, Jaiswal et al. reported the regulatory role of the ERK pathway in hBMSCs osteogenic precursor commitment and differentiation [32]. However, conflicting results obtained from other investigations indicated whether activation of ERK signaling promotes stem cell osteogenic differentiation is a cell-type specific manner [32–36]. In this study, we intend to reveal the importance of MAPK signaling, especially the ERK pathway, in hUCMSC osteogenic differentiation.

## 2. Materials and Methods

### 2.1. Preparation of Human Umbilical Cord Mesenchymal Stem Cells

This study was ethically approved by the Xi'an Jiaotong University IRB. hUCMSCs were isolated and characterized in the manner previously described in the protocol [37]. Briefly, 15 cm long umbilical cord was rinsed with phosphate buffered saline (PBS) and cut into 1 mm^3^ pieces and then digested with 0.1% type I collagenase (Sigma-Aldrich, USA) for 7–10 hours to form a homogeneous gelatinous solution. The gelatinous tissue solution was then mixed with 0.25% trypsin (Gibco, USA) at a ratio of 1 : 1 and incubated at 37°C for 30 min before being diluted in sterile PBS at a ratio of 1 : 10. After being centrifuged at 1200 rpm for 5 min, isolated hUCMSCs were resuspended in a maintenance medium consisting of DMEM/F12 (Hyclone, GE Healthcare Life Sciences, USA) supplemented with 15% fetal bovine serum (FBS; Hyclone, GE Healthcare Life Sciences, USA) and 1% penicillin and streptomycin (Gibco, USA) and seeded in cell culture dishes at a density of 1 × 10^4^ cells/cm^2^. Passage 3 hUCMSCs were characterized as CD90^+^/CD105^+^/HLA-ABC^+^/CD34^−^/CD45^−^/CD19^−^/CD86^−^/HLA-DR^−^ by cell flow cytometry [37]. The differentiation capacity of passage 3 hUCMSCs towards osteogenic, adipogenic, and chondrogenic lineages was verified accordingly [37].

### 2.2. Osteogenic Differentiation Induction of hUCMSCs

Passage 3 hUCMSCs were plated at 5 × 10^4^/cm^2^ and cultured in an osteogenic differentiation medium consisting of DMEM/F12 medium supplemented with 10% FBS, 10 nM dexamethasone (Sigma-Aldrich, USA), 10 mM *β*-glycerophosphate (Sigma-Aldrich, USA), and 50 *μ*g/mL vitamin C (Sigma-Aldrich, USA) for 21 days. Medium was changed every three days.

### 2.3. Inhibit the Activation of ERK by U0126

To block the activation of ERK signaling, 25 *μ*M U0126 (Calbiochem, Merck Millipore, USA), a specific inhibitor of ERK activation [38], was added to the osteogenic differentiation medium for the entire 21-day differentiation period. In a separate recovery experiment, hUCMSCs were only treated with 25 *μ*M U0126 for the first 9 days, followed by a continual cultivation in osteogenic differentiation medium without U0126 until day 21.

### 2.4. Western Blot Analysis

30 *μ*g of protein lysates from hUCMSCs at days 0, 5, 9, 13, 17, and 21 were injected to 10% SDS-PAGE and transferred to PVDF membranes (Merck Millipore, USA), respectively. After blocking with 3% bovine serum albumin (BSA; Sigma-Aldrich, USA), membranes were probed with anti-phospho-ERK1/2 (1 : 500, Santa Cruz, CA, USA), anti-ERK1/2 (1 : 400, BIOSS, China), anti-phospho-JNK1 (1 : 500, Santa Cruz, CA, USA), anti-JNK1 (1 : 500, Santa Cruz, CA, USA), anti-phospho-p38 (1 : 100, Santa Cruz, CA, USA), anti-p38 (1 : 250, Santa Cruz, CA, USA), or anti-GAPDH (1 : 500, Santa Cruz, CA, USA) antibodies at 4°C overnight. After being washed three times with Tris Buffered Saline with Tween® 20 (TBST), the membranes were incubated with horseradish peroxidase- (HRP-) conjugated secondary antibodies (Donkey anti-Rabbit, 1 : 50,000, Abcam, USA, or Donkey anti-Mouse, 1 : 20000, Abcam, USA) at 25°C for 1 hour and developed with commercially available enhanced chemiluminescence reagent (Pioneer, China). Band intensities were determined using ImageJ software.

### 2.5. Quantitative Real-Time PCR

Total RNA were isolated by TRIzol® Reagent (Invitrogen, Life Technologies, Carlsbad, CA, USA) followed by DNase treatment (Invitrogen, Life Technologies, Carlsbad, CA, USA). 1 *μ*g RNA was used for reverse transcription with the SuperScript II Reverse Transcriptase Kit (Invitrogen, Life Technologies, Carlsbad, CA, USA) per the manufacturer's instruction. Real-time PCR was performed on the 7500 Real-Time PCR system (Applied Biosystems, USA) with Maxima® SYBR Green/ROX qPCR Master Mix (Fermentas, USA). Primers used in this study are listed in Table 1. Concomitant *β-actin* was evaluated in separate tubes for each RT reaction as a housekeeping standard. Relative gene expression was analyzed by ΔΔCT method [39].

### 2.6. Alkaline Phosphates Activity

Culture medium was collected and stored at −80°C until analysis. ALP activity in the culture medium was detected by a commercially available kit (Jiancheng biochemical, China) following the manufacturer's instructions. Briefly, each 30 *μ*L culture medium was mixed with 500 *μ*L buffer solution and 500 *μ*L basic solution and then incubated at 37°C for 15 min with standards and a blank. After the incubation, 1500 *μ*L of the chromogenic agent was added to each sample. The absorbance at 520 nm was measured (OD value).

Additionally, ALP staining was performed as previously described [40]. In brief, hUCMSCs were fixed with an ice-cold 60% acetone-40% citrate solution and stained with diazonium salt with 4% naphthol AS-MX phosphate alkaline solution (Sigma-Aldrich, USA).

### 2.7. Alizarin Red Staining

After 21 days of cultivation, hUCMSCs were fixed with 4% paraformaldehyde (Sigma-Aldrich, USA) for 15 min, washed with distilled water, and then stained with alizarin red solution (1% alizarin red and 2% ethanol in distilled water) for 15 min at room temperature. Excess stain was removed by washing with distilled water several times prior to photography.

### 2.8. Immunocytochemistry

After 21 days of cultivation, cells were fixed with 4% paraformaldehyde (Sigma-Aldrich, USA) for 15 min and then washed with distilled water. After blocking with 3% BSA, the cells were incubated with anti-Osteocalcin (1 : 200, Santa Cruz, CA, USA) or anti-Osteopontin (1 : 100, Santa Cruz, CA, USA) antibodies at 4°C overnight. After being washed three times with PBS, the cells were incubated with FITC-conjugated Donkey anti-Rabbit secondary antibody (1 : 10,000, Abcam, USA) at 25°C for 1 hour. Cells were counterstained with 4′,6-diamidino-2-phenylindole (DAPI; Sigma-Aldrich, USA).

### 2.9. Imaging and Image Processing

Images were acquired at room temperature with the CellSens software (Olympus, America Inc., Center Valley, PA) on a fluorescence microscope (Olympus, America Inc., Center Valley, PA) using a 20x (dry HC Plan Apochromat, NA 0.17) objective lens.

### 2.10. Statistical Analysis

All experiments were performed for a minimum of six times. Statistical analysis was computed by SPSS 13.0 (IBM, USA). Statistical comparisons were performed using factorial analysis of variance (ANOVA), followed by an LSD test for comparing treatments between each two groups, and a *p* value less than 0.05 was considered statistically significant. Individual comparisons between two groups were determined by the Mann-Whitney test for nonparametric data.

## 3. Results

### 3.1. Osteogenic Differentiation of hUCMSCs

**Figure 1:**
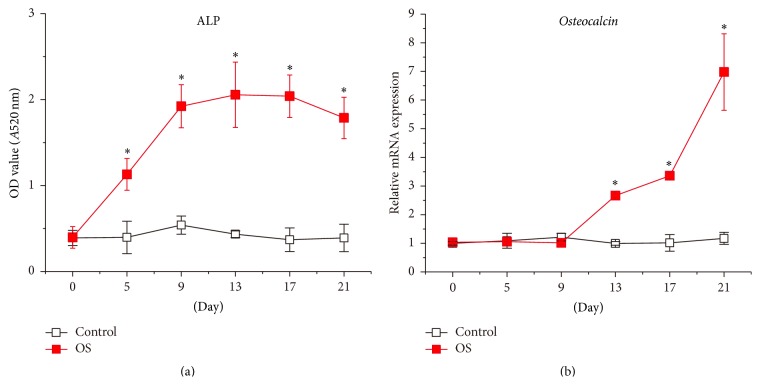
The osteogenic differentiation medium stimulates hUCMSC osteogenic differentiation. ALP activity (a) and transcription of* Osteocalcin* (b) were monitored in hUCMSCs during the 21-day cultivation in either the maintenance medium (Control) or the osteogenic differentiation medium (OS). Data were presented as Mean + SD; ^*∗*^ *p* < 0.05 (*N* = 6).

During the 21-day cultivation in the maintenance medium (Control group), hUCMSCs presented consistent levels of ALP activity (an early osteogenic commitment indicator) (Figure 1(a)) as well as transcription of* Osteocalcin* (a terminal osteogenesis marker) (Figure 1(b)). On the contrary, ALP activity of hUCMSCs cultured in the osteogenic differentiation medium (Osteogenic Stimulation (OS) group) significantly increased at day 5, peaked at day 9, and remained at high levels afterwards (Figure 1(a)). Meanwhile, gene expression of* Osteocalcin* of hUCMSCs continually increased in the OS group from day 9 to day 21 (Figure 1(b)). These data demonstrate that hUCMSCs have the capability of osteogenic differentiation; however, without suitable stimulation such as the osteogenic differentiation medium, hUCMSCs do not go through osteogenic differentiation spontaneously.

### 3.2. Diverse Activation of MAPK Signals during hUCMSC Osteogenic Differentiation

**Figure 2:**
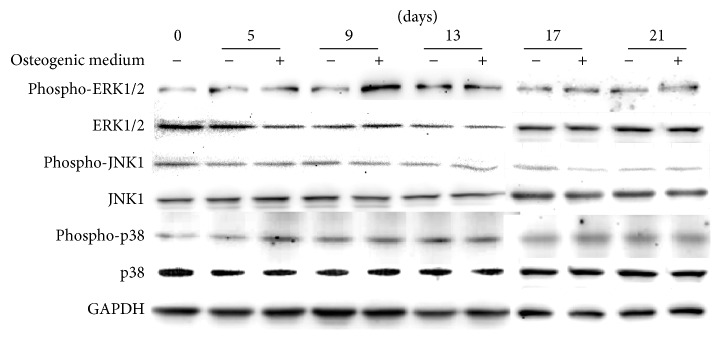
Western blotting revealed that the activation of MAPK signaling pathways was different during hUCMSC osteogenic differentiation. hUCMSCs were cultured in either the maintenance medium (−) or the osteogenic differentiation medium (+).

There are three major MAPKs in mammalians: ERK, JNK, and p38. Although all these three MAPKs are regulated by phosphorylation cascades [41], they may function differently in specific events [30, 31]. Particularly, during hUCMSCs osteogenic differentiation, activation of JNK and p38 was not induced by the osteogenic differentiation medium throughout the entire 21-day cultivation (Figure 2). This suggests that JNK and p38 signaling may not be essential for hUCMSC osteogenic differentiation. On the other hand, phosphorylation of ERK in hUCMSCs robustly increased from day 5 and peaked on day 9, followed by a decline stage thereafter (Figure 2).

### 3.3. Blocking hUCMSC Osteogenic Differentiation by ERK Activation Inhibitor

**Figure 3:**
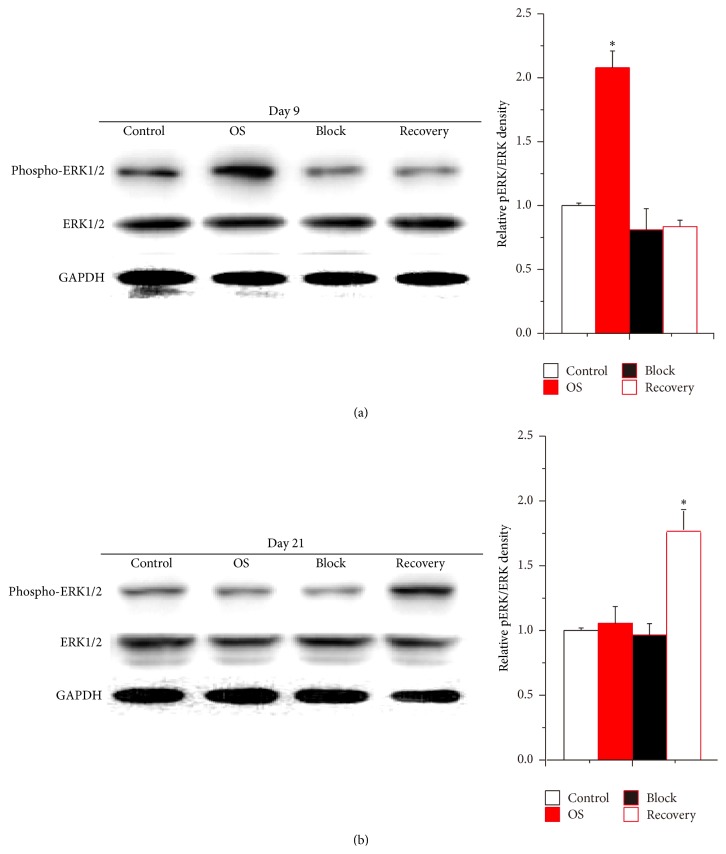
The activation of ERK signaling in hUCMSCs was blocked by U0126. Treated hUCMSCs with U0126 for 9 days completely prohibited the osteogenic differentiation medium-induced increase of ERK activation (a). After the inhibitor was removed, ERK activation of hUCMSCs of Recovery group was upregulated at day 21 (b). The relative densities were normalized to the Control group. Data were presented as Mean + SD; ^*∗*^ *p* < 0.05 (*N* = 6).

To reveal the significance of ERK activation in hUCMSC osteogenic differentiation, U0126, an inhibitor used to prevent ERK activation in BMSCs [42, 43], was added to the osteogenic differentiation medium through the entire 21-day cultivation period (Block group). In this loss-of-function evaluation, U0126 completely eliminated the stimulation of the osteogenic differentiation medium on ERK activation in hUCMSCs (Figure 3).

**Figure 4:**
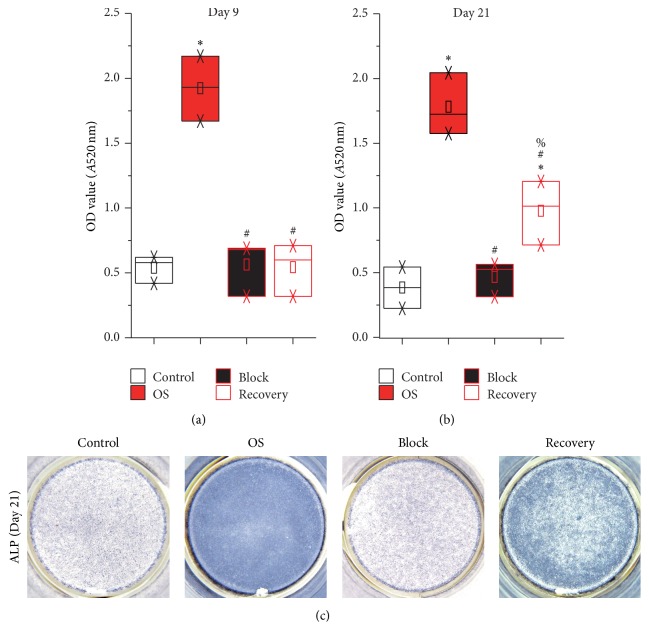
ALP activity was partially rescued in the Recovery group hUCMSCs at day 21. The secreted ALP activities in the hUCMSC culture media were analyzed at days 9 (a) and 21 (b). In addition, ALP staining of hUCMSCs was documented at day 21 (c). ^*∗*^ *p* < 0.05 compared with the Control group; ^#^ *p* < 0.05 compared with the OS group; ^%^ *p* < 0.05 compared with the Block group (*N* = 6).

**Figure 5:**
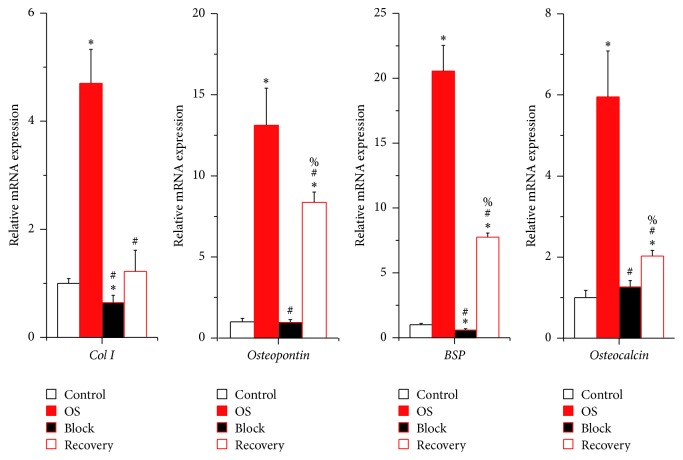
Expression of osteogenic marker genes in hUCMSCs was partially recovered by removing the ERK activation inhibitor U0126 at day 21. Data were presented as Mean + SD; ^*∗*^ *p* < 0.05 compared with the Control group; ^#^ *p* < 0.05 compared with the OS group; ^%^ *p* < 0.05 compared with the Block group (*N* = 6).

**Figure 6:**
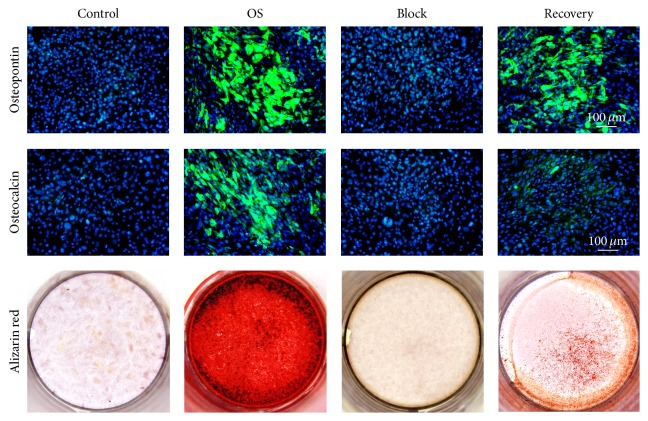
Immunocytochemistry staining of Osteopontin and Osteocalcin as well as the alizarin red staining confirmed the osteogenic differentiation of hUCMSCs in the Recovery group at day 21.

As previously shown, ALP activity of hUCMSCs increased in the culture of osteogenic differentiation medium, which was completely prohibited by continuous inhibition of ERK activation by U0126 (Figure 4). In addition, transcription of osteogenic marker genes, such as* Type I Collagen*,* Osteopontin, Bone Sialoprotein*, and* Osteocalcin*, was also significantly induced in hUCMSCs by the osteogenic differentiation medium (Figure 5). However, this induction was fully abolished by continuous U0126 administration (Figure 5). Immunostaining against Osteopontin and Osteocalcin as well as Alizarin red staining for calcium deposition confirmed the inhibitory effects of the ERK activation inhibitor U0126 on hUCMSC osteogenic differentiation (Figure 6).

### 3.4. Rescuing hUCMSCs Osteogenic Differentiation by Removing U0126

A separate Recovery group, in which U0126 was removed from the osteogenic differentiation medium at day 9 of the cultivation, was employed to further confirm the importance of ERK activation in hUCMSC osteogenic differentiation. Western blotting showed that although ERK activation in hUCMSCs was effectively blocked by U0126 at day 9 (Figure 3(a)), the phosphorylation of ERK was induced by the osteogenic differentiation medium after removing the inhibitor in the Recovery group (Figure 3(b)).

Functionally, the activity of secreted ALP in the Recovery group was significantly higher than those of the Control or Block groups at the end of cultivation, even though it was not comparable to that of the OS group yet (Figure 4(b)). Similar trends were also detected in attached hUCMSCs by ALP staining (Figure 4(c)). Real-time PCR and immunostaining also showed the increase of osteogenic markers expression in the Recovery group at day 21, which indicated the osteogenic differentiation of hUCMSCs (Figures 5 and 6). However, the differentiation of the Recovery group was only partially characterized by lower levels of the osteogenic markers and less calcium accumulation than those of the OS group at the end of cultivation (Figures 5 and 6).

## 4. Discussion

Since its discovery, the MAPK family has been found to play important roles in controlling cellular behaviors. This includes, but is not limited to, cell differentiation induced by intracellular or extracellular stimulation [30, 41, 44, 45]. The subsets of MAPKs are characterized in mammalians: ERK, JNK, and p38 [30]. Although all three MAPK subsets are regulated by phosphorylation cascades [41], they may function independently and distinctly [30, 31]. Previous studies indicate that p38 signaling is involved in stem cell neurogenic, adipogenic, and chondrogenic differentiation. In regard to osteogenesis, the enhancing role of p38 activation was widely described in mouse preosteoblastic cell line [46–49], mouse muscle-derived stem cells [50], human adipose-derived stem cells [33], and both mouse and human BMSCs [32, 43, 51]. Interestingly, since the p38 activation was not upregulated during the osteogenic differentiation of hUCMSCs, our current study implies that the p38 pathway may not be involved in this procedure.

With the osteogenic differentiation medium stimulation, JNK activation was found in the later stage of hBMSC osteogenic differentiation [32]. In addition, studies using a mouse preosteoblastic cell line suggested constitutive activation of JNK increased bone morphogenetic protein (BMP) 2-induced osteoblast differentiation and mineralization [52]. However, Sullivan et al. reported that the JNK inhibitor enhanced osteogenesis in neurofibromatosis type 1- (NF1-) deficient mouse osteoprogenitor cells, including primary neonatal calvarial cells and BMSCs [53]. Moreover, Doan et al. also described the repression effect of JNK on mouse BMSC osteogenic differentiation [43]. However, despite the conflicting observations in the influence of JNK on BMSC osteogenic differentiation, our data suggests that JNK signaling is not critical for hUCMSC osteogenic differentiation.

Meanwhile, the negative impact of ERK signaling on osteogenesis was also observed in the mouse preosteoblastic cell line [49, 54] and hBMSCs [43]. Actually, constitutive increases in activated ERK signaling were recognized as the reason for impaired osteogenesis in NF1-deficient patients [36]. Moreover, the blockade of the ERK activation in* Nf1* ^−*/*−^ mBMSCs could attenuate the increased cortical porosity observed in mutant pups [36, 55]. Conversely, in other studies, activation of ERK was thought to benefit mBMSC [33] and hBMSC [32, 45, 56] osteogenic differentiation. In this study, we found that the osteogenic differentiation medium strongly activated ERK, but not JNK and p38, in a time-dependent manner in hUCMSCs. By employing loss-of-function and recovery studies, we further confirmed that the activation of the ERK pathway critically regulates the osteogenic differentiation of hUCMSCs, another example of how hUCMSCs are not identical to hBMSCs [16, 17]. This discovery enriched our knowledge of underlying mechanisms behind the regulation of hUCMSC osteogenic differentiation and set up fundamental ideas to more effectively stimulate hUCMSCs conversion towards osteogenic lineage in cell therapy and tissue engineering strategy.

It is worth noting that several diverse techniques have been developed for the dissociation of tissues for primary cell isolation. To obtain hUCMSCs with high quantity and high stemness, especially with a higher capacity for osteogenic differentiation, a previously described collagenase/trypsin-based isolation method was used in this study [37, 57, 58]. Since Salehinejad et al. revealed that the isolation method could profoundly alter the cell harvesting and proliferation by comparing different methods for hUCMSC isolation from human umbilical cord Wharton's jelly [57], the osteogenic potential of our hUCMSCs used in this study was slightly different form that of the hUCMSCs reported by Bosch et al. [22].

In summary, our current results demonstrated that the activation of the ERK signaling pathway, but not JNK or p38, was necessary for the osteogenic differentiation of hUCMSCs, which deepened the understanding of the nature of hUCMSCs, a relatively new alternative stem cell source for tissue engineering. Moreover, our study significantly benefits the application of hUCMSCs, particularly in bone tissue engineering, by pointing out a potential regulatory direction to stimulate hUCMSC osteogenesis for engineered bone tissue generation.

## Figures and Tables

**Table 1:** List of primers for quantitative real-time PCR.

Gene	Sequence
*β-actin*	5′-ATC GTG CGT GAC ATT AAG GAG AAG-3′
	5′-AGG AAG GAA GGC TGG AAG AGT G-3′

*Collagen type I, α1 (COL 1A1)*	5′-GTG AGA CAG GCG AAC AGG-3′
	5′-GAC CAG CAG GAC CAG AGG-3′

*Osteocalcin (OCN)*	5′-ACA CTC CTC GCC CTA TTG-3′
	5′-CAG CCA TTG ATA CAG GTA GC-3′

*Osteopontin (OPN)*	5′-GAA GTT TCG CAG ACC TGA CAT-3′
	5′-GTA TGC ACC ATT CAA CTC CTC G-3′

*Bone Sialoprotein (BSP)*	5′-CCC CAC CTT TTG GGA AAA CCA-3′
	5′-TCC CCG TTC TCA CTT TCA TAG AT-3′
